# Behavioral profiles and social relationships in Wiedemann–Steiner syndrome: parent reports on 25 cases

**DOI:** 10.1186/s13023-025-03643-1

**Published:** 2025-04-02

**Authors:** Nicola Yuill, Camilla Elphick, Jess Marshall, Wendy D. Jones, Jane Waite, Hannah Viner

**Affiliations:** 1 School of Psychology, University of Sussex, Pevensey Building, Brighton, BN1 9QH UK; 2 School of Psychology and Counselling, The Open University, Milton Keynes, UK; 3 North East Thames Regional Genetics Service, Great Ormond Street Hospital for Children, London, UK; 4 School of Psychology, Aston University, Birmingham, UK

**Keywords:** Wiedemann–Steiner syndrome, Autism, Social relationships, Behavioral profile, Genetic disorders, *KMT2A*

## Abstract

**Background:**

Wiedemann–Steiner syndrome (WSS) is a rare, variable neurodevelopmental condition associated with developmental delay, intellectual disability and congenital abnormalities. There are few investigations into behavioral characteristics. Importantly, parental perspectives are particularly lacking. This study investigated commonalities in the behavioral characteristics through the perspectives of parents’ lived experiences.

**Method:**

We conducted in-depth interviews with 25 parents of children with WSS in the United States and United Kingdom, tapping lived experience and specific examples of behavior, relationships and communication. Responses were analysed using reflexive thematic analysis.

**Results:**

We report three main themes: intense sociability (confirming questionnaire-based research), intense relationships and executive dysregulation (novel findings). We also found previously unreported sensory sensitivities and cognitive patterns of uneven memory and poor comprehension.

**Conclusions:**

These data direct from parent experience reveal novel commonalities in behavior and relationships in this group. Findings should inform clinical assessment and diagnosis, new research questions and choice of patient-focused outcome measures for clinical interventions. The findings also contribute to improved practice in providing care and support for people with WSS and their families and to guidelines for more tailored education and improved healthcare.

## Background

Wiedemann–Steiner Syndrome (WSS) is a rare neurodevelopmental condition with estimated prevalence 1 in 25,000–40,000 [12]. WSS was first reported by Wiedemann and Steiner in 1989 and 2000 respectively [27]. WSS is a variable disorder generally including developmental delay, short stature, distinctive facial features, feeding and digestive difficulties, among other differences (33 cases reviewed by [2], 104 cases by [26]). Physical and mental health difficulties are also reported, notably anxiety [16]. WSS is caused by pathogenic variants in *KMT2A* (formerly known as *MLL*), which encodes a histone methyltransferace [13]. Pathogenic *KMT2A* variants are also observed in large cohorts of individuals with conditions such as autism [5] and epilepsy [1, 10].

Since the genetic cause was uncovered, clinical research and identification of affected individuals has increased significantly [17]. However, very little of this work investigates the behavioral and social features of WSS, in comparison to research into genetic and medical aspects. For example, Yu et al. identified 248 reported cases, categorised primarily by medical and physiological factors. Behavioral aspects covered just three factors: 211/217 (97%) people were reported as having intellectual disability, 78/88 (89%) with speech delay and 74/183 (40%) with the very broad label of ‘behavioral disorder’. A similar catch-all category was reported in 32–42% of cases by Baer et al. [2]. Durand et al. [6] found 8 of 21 case reports of people with WSS meeting criteria for Attention Deficit/Hyperactivity Disorder (AD/HD) diagnosis and 5 of the 21 meeting criteria for an autism spectrum condition (ASC). Anxiety has been cited in self-reports of two-thirds of 18 participants with WSS [18] and in 8 of 21 developmental histories in people with WSS [6]. Crucially, Ng et al. [16], in a study of 24 parents completing questionnaires, have recently identified social features of WSS in relation to autism, notably autistic traits such as inflexibility, together with high social drive. Otherwise, there is little work focused specifically on behavioral aspects of the condition that inevitably affect the everyday lives, social relationships, care and support of such children and their parents and carers.

Different neurodevelopmental conditions can show distinct profiles of behavior that affect relationships and support needs, but patterns can also be shared across diagnostic boundaries [25]. In particular, social behaviour—how children reportedly engage with their social worlds—has a powerful impact on communication, relationships and hence the design of support. Patterns can vary widely from hyper-sociability in Williams’ syndrome, or frequent laughing in adults’ presence in Angelman syndrome, to reduced or atypical social engagement in autism. Anecdotal reports from parents suggested high sociability in WSS, seemingly contrasting with concurrent ASC diagnoses in some WSS cases. By asking parents about the general behavior of their WSS children we aimed to elicit more clarity on patterns of social behavior.

Parents face significant challenges in supporting their children’s physical health needs, and some research is rightly focused on this area. However, anecdotally parents also cite the need for support and professional knowledge in understanding and managing behavior, relationships and education. This is a particular challenge, not only because of physical health needs requiring support, but also because of the lack of knowledge about rare disorders such as WSS in education and healthcare. Behavior support needs can differ markedly across different genetic syndromes [23, 29], making syndrome-specific research crucial for providing suitable recommendations. Our first main aim is to undertake a more differentiated and precise examination of behavior and relationships in WSS than has been reported in research to date.

A second need for parents is having their own everyday experiences heard as a starting point for research. Existing studies generally use standard measures chosen by the researchers, primarily oriented towards more-researched neurodevelopmental conditions such as autism and AD/HD. There are crucial psychometric reasons for using pre-existing scales, but this approach is usefully complemented by investigating common behavior patterns based on parents’ own words and experiences.

Understanding behavioral and social aspects is crucial for parents and professionals in addressing the social relationships, everyday care and educational needs of children and young people with WSS, as well as for raising questions for further research. This complements approaches that start from existing diagnostic categories and clinical scales. Our second aim was to start from parental experiences to identify such patterns in WSS, to inform future research and to support practical help.

## Method

### Design

Parents of children and young people with WSS were the primary informants, using a relatively broad open-ended interview frame (see Appendix 1) addressing behaviour, communication and relationships and schooling, given our interest in mapping support needs, with a final question on strengths. We encouraged parents to give examples and sought further depth based on parents’ responses.

The research was initiated when Author2 was approached by the WSS UK foundation through a personal contact about the general research question of how parents might be better informed and supported to help their children. Authors 1&2 who respectively had research and lived experience in developmental disabilities, consulted Author4, a clinical geneticist working with WSS, about behavioral challenges encountered and attended a WSS information day, involving hearing parents’ concerns and questions. The research design and interview protocol were approved by the University of Sussex Ethics Committee, ref ER/JM795.

### Participants

Parents were recruited by advertising through WSS foundations, including Facebook groups in the United Kingdom (UK) and United States (US). Twenty-five parents of children with WSS (23 mothers, 2 fathers; 14 UK, 11 US; parents of 11 sons and 14 daughters with WSS, aged 2–32 years, mean age 8 years) agreed to an interview. Other demographic information was not sought. An additional parent living outside UK/ North America was not included as the data protection protocol did not include their country of residence. Age bands of the affected child are shown in Table 1. All had a diagnosis of WSS. Parents of nine children reported additional diagnoses: two females and one male with a diagnosis of autism, two males and two females with a diagnosis of AD/HD (one ‘informal’/ referred), one female with anxiety and one female with diagnoses of both ASC and AD/HD.

**Table 1:** Number and percentage of male and female WSS children in each age band

Age band	Female (%)	Male (%)	Total
0–5	7 (70)	3 (30)	10
6–10	2 (33)	4 (67)	6
11–15	2 (33)	4 (67)	6
16–20	2 (100)	0 (0)	2
21 +	1 (100)	0 (0)	1
Total	14	11	25

### Procedure

We emailed parents information and consent forms, then arranged a telephone interview between June–September 2019, with interviews recorded on a secure digital audio device. The interviews lasted 40–90 min and were conducted by Author3 (17 interviews) and Author6 (8 interviews), who co-trained on the schedule instructed by Author2, ensuring consistency of approach. The interviewers had previous practical experience and education in neurodisability and attended an informal WSS family event, meeting children and hearing parents’ concerns, aiding them to interview with genuine curiosity and empathic interest.

The interview began with an overview of purpose and reminder of withdrawal rights, then a further verbal consent. Interviewers noted that sensitive topics might be raised, reminded parents of available support and right to withdraw: no parent withdrew. The interview closed with discussion of positive aspects in life with the child, to serve a balancing function for interviews that may have focused on difficulties. Finally participants were thanked for their participation. Several participants expressed gratitude for and benefit from the opportunity to talk about their experiences.

### Materials

The interview (Appendix 1) had three main sections created after consultation by Author1 and Author2 at the WSS parent information day. It addressed (1) behavior and relationships (reported in this paper) (2) experiences of diagnosis and (3) priorities and needs for further research. Questions were intended to support parents to describe in their own words and to lead as little as possible, e.g.: ‘This is a broad question: can you describe [child’s] behavior in general?’ with open-ended follow-up, e.g. ‘Can you tell me more about that/ give an example?’, or repeating a parent statement and asking for expansion, e.g., ‘You said his behavior at school was different: Can you tell me more about that/give an example?’.

### Data treatment

Audio was stored on a secure server and interviews securely transcribed by a team of three people independent of the current authors, using an ‘intelligent verbatim’ method [15]. This method enables removal of disruptions to the call/ side-comments, but includes all relevant utterances, repetitions and hesitations. Identifying information was redacted as far as possible from the transcribed interviews, which were stored on a secure server. Author1 extracted all text relevant to children’s behavior. This was primarily responses to the relevant questions in Sect. "Background". Infrequently, where a parent returned to a Sect. "Background" topic in Sects. "Method" or "Results", as judged by Author1, that comment was added to the transcript for the current analysis.

Transcripts were coded inductively and analysed using a reflective thematic analysis approach (Clarke and Braun 2021). Author1 and Author3 each made notes and initial groupings independently, generating 14 and 16 codes respectively. These groupings and extracts from the data to support them were then shared and discussed with Authors 2 and 6. Following these discussions Author1 generated the final revised set of 12 codes, combining where codes overlapped and deleting to avoid overlap, and coded all the transcripts using the amended themes. The labels and descriptions of the themes were tweaked, in light of examples from the data, in discussion with all authors, with Author4 and Author5 providing expertise respectively in clinical genetics of WSS and in clinical and research on rare syndromes and intellectual disability.

## Results

The analysis resulted in four main themes, each containing three subthemes (see Table 2). Themes and subthemes are labelled in ways that represent best the organising principle used in coding, and include a typical parent quote for each subtheme.

**Table 2:** Themes, subthemes and parent quotes

Theme and subthemes	Sample quotes	N (%) cases
1 Intense sociability		
1a Unboundedly social	‘everyone’s friend’; ‘he loves …the attention…life and soul of the party’; ‘extremely well-liked kid… he is interested in other people’; ‘socially he can be over the top’; ‘makes friends very easily, speaks to anyone…doesn’t understand what strangers are’	19 (76%)
1b Intensely affectionate	‘sticks to me like glue’; ‘she likes to give people kisses … like a giant lick on the face’; ‘he cuddles and hugs …his friends…they don’t really want it’	13 (52%)
1c Capacity for positivity	‘a happy kid’; ‘she’s happy all the time’; ‘he really is just a happy kid’; ‘she’s really happy… just very joyful and it’s really contagious’; ‘a light from within that draws people’	8 (32%)
2 Intense/exclusive relationships		
2a Prefers adults	‘I call her Velcro next to me’;’She will prefer to bond with adults rather than children’; ‘she’s like addicted to me’	11^1^ (48%)
2b Intense sibling relations	‘a love-hate relationship’; ‘has a very love-hate relationship’ ‘it’s a hot and cold situation… they [siblings] understand how to press on each other’s bruises’; ‘she can be extremely loving towards her sister and then other times she deliberately pushes her away’	9^2^ (47%)
2c Younger play style	‘plays with younger kids’; ‘she plays, either alone or be the boss of what she’s playing’; ‘children of his age …have different interests ‘	8^1^ (35%)
3 Dysregulation		
3a Inflexibility	‘change is a big thing for her. She hates change’; ‘if she wants something it’s really difficult to actually dissuade her’; ‘she cannot tolerate change’	20 (80%)
3b Impulsiveness	‘he can go from like 0–10 in 5 s’; ‘zero to full-blown in the blink of an eye’; ‘like a flick of a switch where he just gets really cross’; ‘she has no fear’; ‘he won’t look when crossing a road, he’ll just run straight off’	16 (64%)
3c Aggression	‘very physical’; ‘hitting, biting, pinching, a massive issue’; ‘he will hit out, he will kick, he …has bitten’; ‘pinching and shouting and pushing …a lot of physical aggression’	18 (72%)
4 Sensory and cognitive patterns		
4a Sensory sensitivity	‘loves crazy loud noises’; ‘she does like…being able to squish things’; ‘a lot of sensory issues, like brushing her hair’; ‘she hears things like a few seconds before anybody else’; ‘a heightened sense of smell… likes certain feels… and the taste of certain textures … that she doesn’t like’	16 (64%)
4b Comprehension gaps	‘reading is his strength … understanding is a bit iffy but the pleasure he gets from it’; ‘she can read a book really well.. at least on par with her peers, but if you ask her what’s happened …she can’t tell you’; ‘he can read very long big words but if you give him a paragraph to read… ask him a question, he probably wouldn’t be able to answer it’	7^3^ (39%)
4c Uneven memory	‘he has the most phenomenal memory’; ‘we drove down a street that we’d only driven down once in our lives, and he remembered immediately who lived there …[a smell elicited memory of] exactly who was sitting where, who ate what, who drank what’; ‘she’ll sometimes refer to a slide [from] three years ago as if it was yesterday; ‘she really remembers names… she remembers places’	4^3^ (22%)

N = 25 unless otherwise specified, where cases were excluded for questions that were not age-appropriate. 1: *n* = 23, excluding 2 infants. 2: *n* = 19, excluding children with no siblings. 3: *n* = 18, excluding children primarily non-verbal

### Theme 1: intense sociability

This theme appeared widely across the age range, from 2 to 32 years, and included two of the four children with an autism diagnosis. Only three children, two of those autistic, had no responses fitting this theme.

A striking subtheme within this was *1a, sociability without boundaries*: parents often mentioned this as the first description given and often with similar phrasing.

This subtheme included both strong interest in people and positive responses from others. Parents saw the great benefits of this, together with risks from the high degree of openness, trust and unwariness, potentially creating social vulnerability.

Responses in *Subtheme 1b, intense affection,* were generally expressed in very physical ways, sometimes together with intense sociability. Parents mentioned both the affection involved in the physicality, and the reactions from others: peers in particular, could find such expression a violation of personal space. Twelve of 13 responses here also cited subtheme 1a.

*Subtheme 1c, capacity for positivity*, cited a generally happy mood, sometimes contagious. These cases all concerned children under 4, except for 1 11-year-old.

### Theme 2 intense and exclusive relationships

This theme was divided into three *Subthemes: preference for adults, intensity of sibling relationships and play patterns typical of younger children*. The two youngest children (2 years of age) were excluded at the outset of selections for this theme, because their age sufficiently explained a close bond with the main carer and the restricted possibilities of play with peers or siblings.

Parents reported their children (aged from 5 to 32) had a *strong preference for adults (Subtheme 2a)*. In eight cases there was a particular desire for closeness with or strong dependence on the mother, and in three of those, parents mentioned anxiety as a clear factor. This Subtheme was not strongly associated with presence of Subtheme 1b, intense physical affection.

*Subtheme 2b, intensity of sibling relationships* was examined in the 19 of 23 cases coded who had siblings (also excluding the two infants as noted above). In two cases these were half-siblings that children saw or shared space with regularly. Sibling relationships were often described as intense.

Increasing gaps in mental maturity could create challenge to the sibling relationship:

‘as they [younger sibs] got older they started overtaking him; he’s found that hard’.

These relationships could also be affected by volatile behavior of a WSS child (see also Theme 3 below).

There were examples of siblings playing caring roles for the WSS child and successfully managing difficult behavior:

‘she’s usually able to go in there and calm him down’.

*Subtheme 2c*, *play patterns typical of younger children*, covers interactions reported across peers and/or siblings. Parents noted that play or socialising opportunities with peers could present difficulties for some children and young people. Some school-age children were described as playing alongside rather than with peers, and for a partly overlapping set of school-age children, there was stated preference to play or get along better with younger children. As parents explained, this might arise from immaturity in play style, possibly related to the child’s lower intellectual level and younger interests.

However, for some children, peer play still provided opportunities that appeared satisfying:

‘he doesn’t realize he’s not really playing their game and every now and again, somebody will just kick the ball back to him and he thinks he’s playing football with the big kids’.

At least two children reportedly established warm and supportive friendships outside the family:

‘the three of them [WSS child and friends] just like play so harmoniously’

‘the senior year she started to make friends’.

### *Theme 3 dysregulation*^1^

This theme addressed behaviors involving lack of inhibition, impulsiveness and physical aggression. *Subtheme 3a, inflexibility*, especially experiencing difficulty with transitions, was common.

An important facet of this was parents’ experience of the benefits of routine:

‘she responds really really well to structure and routine’.

Often, behavioral difficulties were not seen at school, and parents cited routine as important here: this home-school difference caused some strain, with parents feeling schools did not recognise the difficulties:

‘they don’t always see the struggles that we have at home’

and that their children could come home and experience meltdowns after a day of restrained behavior at school. Two parents mentioned ‘masking’, commonly used in respect of autism to describe behavior where children are purposely concealing atypical behavior to fit in. In this context parents were referring to strain caused by maintaining inhibited behavior, such as sitting for long periods, until returning home:

‘it’s exhausting for him to keep the behavior in check like he knows he must so we get the brunt of it, by…4:30’.

*Subtheme 3b, impulsiveness and lack of inhibition*, was also pronounced, with parents citing quick switches in moods and behavior.

This subtheme also included behaviors that were impulsive, and potentially dangerous, with concerns about road safety, running off, or being a ‘daredevil’. The five children with a concurrent reported diagnosis of AD/HD were, as might be expected, all included here.

Several parents also mentioned co-occurring inattention and lack of focus.:

‘her focus is very poor’.

Some cited their difficulty in predicting or controlling their child’s responses:

‘there’s not always a trigger’.

‘if…he doesn’t want to do that, no matter what you do, it doesn’t work’.

and difficulty for the children themselves in explaining their sudden behavior changes:

‘she can’t tell you why she’s upset’.

*Subtheme 3c relates to aggression*, the word often chosen by parents, and reported widely across the full age range. Two cases were restricted to self-injury only.

In line with Subtheme 3b, parents noted they sometimes had difficulty recognising triggers for aggression, though some situations predictably produced it.

The triad of inflexibility, impulsiveness and aggression was commonly found together, with 13 parents (52%) citing all three. Consistent with the experience of high dysregulation in WSS children, mentioned earlier, seven parents reporting aggression were among nine overall who especially noted their child’s rapid remorse and sorrow after aggressive or impulsive behavior:

‘after…he gets very upset… knows that he has hurt you’

‘burst into tears and be very apologetic about his behavior’.

### Theme 4 sensory and cognitive patterns

The final theme differs in that we did not directly ask about sensation and cognition, whereas the previous themes related directly to questions about behavior and relationships. Many responses in this theme arose from the question about children’s communication methods. We split this theme into three Subthemes: *4a, sensory sensitivity, 4b, comprehension gaps and 4c, uneven memory.*

*Subtheme 4a, sensory sensitivities*, was striking. Expressions of sensitivity type were variable and, given their frequency, we categorised them using an autism-derived scheme as sensory-seeking, sensitivity to noise (audio filtering) and sensory reactions in taste and smell [14].

Eleven parents reported their children’s sensory-seeking, and some used techniques such as stroking or squeezing to help children self-regulate:

‘he likes the …the deep pressure’.

‘she is a sensory seeker…you gotta squeeze her…loud music…the bigger the better’.

Five parents reported their child’s highly sensitive reactions to noise (‘auditory filtering’). For three of these five children this was hypersensitivity to sound exclusively: the other two reportedly experienced high sensitivity in other senses.

Five children also showed high sensitivity to taste and/or smell, with four of these showing other sensory traits in addition. The smell/taste group included three of the four children who had also been diagnosed as autistic.

Such sensitivities seemed not closely associated with the strong preference for physical affection: that appeared both in children with and without reported sensory sensitivities.

*Subtheme 4b* involved comprehension gaps. The ‘communication’ interview question revealed wide variety in the sample’s cognitive level, and language use. Seven children (including all three 2-year-olds) were primarily non-verbal, some using sign. Three parents of older, fully-verbal children noted that speech development had been delayed.

Seven parents noted a specific gap between a child’s ease with decoding text and their relatively poor comprehension of language, including one parent whose child showed a large gap in decoding –comprehension on a standardised test.

We also include pragmatic language differences in this subtheme. These included echoing others’ speech, mimicry or use of formulaic language creating a superficial impression of social facility:

‘she holds on to these little nuggets of decorum

‘

‘he will randomly give them information that isn’t relevant’.

Parents also mentioned children taking statements over-literally, disregarding relevance and talking intensively on one topic of great interest, e.g. an intense focus on plane markings:

‘he’ll just randomly give them information that isn’t relevant…or maybe of any interest, has a tendency to interrupt a lot - he gives too much information’.

Parents also gave examples of oversharing:

‘she doesn’t know how to filter anything, so whatever she’s thinking is going to come out of her mouth’.

Two parents reported their children’s enjoyment in language play, inventing words and using words beyond age-typical vocabulary (though not necessarily comprehending).

Subtheme 4c reflected comments about unevenness in memory. In particular, four parents mentioned ‘phenomenal’ and ‘uncanny’ memories from the past.

This vividness of memory was sometimes in the context of particular intense interests, and two parents contrasted this with perceived short-term memory impairments that compromised school achievement.

## Discussion

Our thematic analysis of interviews with 25 parents of children with the rare genetic condition of WSS elicited three main themes in behavior and relationships: intense sociability, intense relationships and dysregulation, with an additional theme of sensory and cognitive differences. This information supports and extends findings derived from clinical diagnostic assessments and standardised questionnaire measures derived from more common conditions. Most previous WSS research focused on physiological and neurocognitive phenomena with limited information about social behavior and relationships. We discuss each theme in turn, raise further research questions and describe limitations and implications for research and practice.

High sociability was almost ubiquitously reported in this sample, often accompanied by intense desire for affection, often physically-expressed and focused on one parent, and a strong capacity for positive emotional expression. This provides convergent evidence with Ng et al. [16] who had 24 parents complete clinical scales that revealed WSS children’s high social motivation and prosocial tendencies. Research examining social motivation in syndromes with similar behavioral profiles, such as Williams and Rubinstein-Taybi syndromes, suggests that social motivation is often preserved in the presence of differences in social cognitive abilities, such as Theory of Mind [8, 11]. High sociability can be positive in encouraging social relations, but if combined with differences in reasoning about others’ intentions, can leave individuals vulnerable to social exploitation. Several respondents expressed fear of negative consequences of their children’s high trust of unfamiliar people.

This high sociability might seem inconsistent with a reported partial overlap with autism [6], and given that we found two of the children coded in this theme also had an autism diagnosis. However, some autistic children can show intense interest in others, and low sociability is not a universal pattern in autism [25]. Chan et al. [4], in a clinical phenotyping study, suggested that 5 of the 6 WSS children they studied may have a subtype of autism characterised by social motivation, inflexibility, emotional dysregulation and externalising behaviors. Machine learning approaches to social profiles in autism and genetic disorders may cast further light on distinct patterns [3]. Possible relations between WSS and autism, particularly in respect of social motivation, deserve further research (see also [19]).

Frequent positive mood was notably reported only in younger children and parents generally cited this as a strength. Longitudinal studies would investigate a potential pattern of lowered mood over development, and possible underlying factors. Pain, commonly experienced in WSS, is a plausible causal factor that could affect mood over time. External demands, such as attending school, or transition to secondary education, might bring added challenges for WSS children, particularly in the need for regulation, and this could potentially increase anxiety.

Accompanying the picture of high sociability was high reported frequency of close, sometimes exclusive, relationships with a caregiver, and a preference for adults over children. Where children had siblings, these relationships were fairly often reported as volatile, both loving and conflicted. Peer relations, possibly overshadowed by close relationships with carers, were sometimes characterised as typical of younger children. A pattern of intense relations with caregivers has been noted in other syndromes, such as Smith-Magenis syndrome [29].

The third theme, dysregulation, covered behaviors reported as inflexible, impulsive and sometimes aggressive. This theme is consistent with previous research that suggests an overlap with diagnostic features of AD/HD [6], hyperactivity and ‘conduct problems’ [19]. In particular, Ng et al. had 24 parents of WSS children complete the Strengths and Difficulties Questionnaire (SDQ: Goodman, 1997), and found scores reached ‘clinically significant’ for the Emotional Problems, Hyperactivity, and Peer Problems categories (each a 3-point Likert scale for 5 items per category). The current study, using a different method of open-ended reports from parents, supports this behavioral picture. Parents’ comments show how these patterns affected everyday life, potentially informing better understanding of the sources of these behaviors and means of supporting children. For example, some parents were concerned that impulsive behavior plus high sociability put their child at risk of harm, and that volatility could affect the close relationships children often had with family and friends.

Parents reported a high level of challenge and elevated levels of volatile and aggressive behavior. The multiple possible factors behind this need further investigation, with a strongly-expressed need for parental support. Parents often noted their children had to cope with physical distress related to the gastro-intestinal problems common in WSS, and to commonly-occurring sleep problems. All children had experienced long and sometimes painful medical interventions in their lives, for diagnosis and/ or physical problems associated with WSS. Further research might address whether unpredictable behavior and emotional dysregulation could relate to internal pain [29]. The combination of executive dysregulation and sensory sensitivities suggest these factors could prompt physical outbursts, particularly if children are unable to communicate readily or control sensory discomforts [22].

Parents did not often mention anxiety, surprising given previous findings. Ng, Bjornsson, et al. [18] reported 9 of 18 WSS participants (aged 8–33 years) had an anxiety diagnosis. However, our finding of intense close relationships and preference for an adult corresponds with the Ng et al., study which found a high prevalence of separation anxiety. The younger average age in the present study, with 16 children under the age of 10, might explain the absence of reported anxiety diagnoses, which may increase post-puberty. Anxiety may manifest differently in children with learning disability, and it may be that parents did not label behaviors as anxious [7]. The studies by Ng and colleagues expressly asked about anxiety, whereas we did not.

We identified a further novel theme of cognitive and sensory differences, although we had not specifically tapped these psychological aspects. The sensory sensitivities that parents mentioned coincides with findings by Durand et al. [6]. Using the Short Sensory Profile, they found 7 of their 33 WSS participants had elevated scores. They also noted the overall frequency of sensory processing differences in children with intellectual disability [9]. These findings suggest further research into patterns of sensory difference that could bring better understanding of behavior patterns and improved recognition of support needs in WSS.

Several parents noted differences in spoken and written communication, especially pragmatic language differences, such as over-sharing information, echoing and using formulaic language, sometimes with enjoyment of language play. For both written and spoken language, some parents suspected children’s speech and reading skill exceeded their comprehension. This is important, because Ng et al. [16] reported high variability in parent-rated reading scores for WSS children, but the questions on the scale used related to decoding (e.g. phonics) rather than comprehension of what is read. Further research should investigate whether WSS children show a pattern of decoding in advance of comprehension, as is found more generally in some children [20, 24]). Strong language play and vocabulary suggests distinguishing between oral and written language skills is important, especially given that some children, owing to chronological and intellectual level, may not have mastered decoding.

Parents occasionally mentioned poor short-term memory but three noted examples of ‘phenomenal’ longer-term memory, for example in relation to remembering names (linked to high sociability and friendliness with strangers) and strong visual and spatial memories from the past, sometimes linked to special interests such as planes or musicals, although we had not asked about these aspects specifically. Ng et al. [16] reported a common pattern of visual and spatial skills below age expectation, using formal memory assessments of immediate and delayed recall. Differentiation between separate aspects of short- and long-term memory skills, including assessing autobiographical memory, and the potential links between memory and social motivation, deserve further investigation. More generally, further research could examine how language, mathematical and spatial skills vary with level of general intellectual ability, and any relation with autistic features such as focus on special interests.

Understanding rare syndromes such as WSS could be amplified in further research by making comparisons with other syndromes. For example, the close attachment to a caregiver or strong social drive for adult attention, which may co-occur with aggression when attention is removed, may have parallels with Smith-Magenis syndrome and Angelman syndrome respectively [21, 30]). The rapid remorse and distress after outbursts might be compared to findings in Prader-Willi syndrome [28].

Cross-syndrome comparison has strengths and weaknesses. Identifying common behavioral patterns reported here could encourage stakeholders to consider techniques used to support children with other conditions who show similar behavioral patterns. For example, many WSS children experience sensory sensitivities and some parents used methods common in conditions such as autism: use of headphones, weighted blankets and calming sensory touch. As sometimes reported in autism, difference in behavior between home and school was often attributed to the routines of school, and some parents had aimed to provide structure at home, where possible, and to use visual timetables and advance preparation to mitigate the difficulty in transitions experienced by many of the WSS children. Parents reported being referred to support for more well-known conditions such as autism and AD/HD. However, some parents in our sample did not find such referral helpful, so translation of advice needs to be carefully considered, with information tailored to experience with WSS. Analysis of parents’ experiences, including diagnostic assessment of their children, shows a deep sense that help and advice is not always available from experts or professionals providing care and education services, since WSS is so little-known and understood: rather, we found parent groups often cited as more helpful and supportive.

The results raise questions for further investigation, which might include the following:

- What benefits and challenges might follow from intense sociability?
- How might preference for adults, or a single adult, relate to anxiety?
- How might behavioral volatility and cognitive and emotional differences from peers affect peer relations?
- What qualities of peer relations provide opportunities to develop?
- How might home-school differences in behavior, and differences in routine, be bridged?
- How can volatility and rapid behavior change be better predicted and managed? Are pain and communication skills a factor?
- Are there discernible patterns in sensory sensitivities and could greater sensory management help?
- How common are pragmatic language difficulties and is there a common decoding/comprehension gap in reading?
- Is there unevenness in different aspects of memory, e.g. visual, autobiographical and for special interests?

The present results are clearly limited by the size of the sample and the lack of demographic information to check representativeness: further studies should investigate the commonality of the themes reported here. Our interviews focused on behavior, which would influence how parents responded. For example, anxiety did not appear as a theme despite its appearance in the research literature: it may have done so if we had phrased our questions about mental health. Some participants will have known each other, given we used parent-support social media for recruitment, so parents may have used similar wording in describing their children. However, parents were encouraged and willing to report specific examples of their child’s behaviors to illustrate each point.

The study is based on parent open-ended report, providing a complement to other studies of WSS using standard clinical scales. Clearly there are sound reasons for using such scales (see [6]) but these can only test what is on the scale. Parent reports cover the lived experience of challenges and strengths of supporting people with WSS and have potential to reveal areas for further research with appropriate standard measures. Our findings support and extend previous research suggesting high sociability (notably, [16]) and close attachment, and further reveal dysregulation patterns and sensory/cognitive differences. Our results also reflect strengths that parents see in their WSS children. Further research crucially needs to be informed by the perspectives of people with WSS themselves: we did not include children’s own perspectives.

## Conclusions

This paper began with parents’ reports of their own experiences of a child with WSS. The themes we derived from the data comprised intense sociability, with a lack of boundaries, intense affection, often physical, and a capacity for positivity which parents valued highly given challenges faced by families in other areas, but that sociability also conferred potential vulnerability. Social relationships were often seen as intense and exclusive, with some preferring adults, sometimes intense sibling relations and a younger play style that could require accommodation by others. Dysregulation was also a common theme, with reduction in flexibility (difficulty in transitions), impulsiveness and sometimes, physical aggression. We also noted specific sensory sensitivities and characteristics affecting cognition, notably comprehension gaps and uneven memory. All of these have implications for everyday adjustments and management to enable children with WSS to thrive.

As well as documenting unmet patient needs through parent voice, this study paves the way for determining patient-focused relevant outcome measures for developing effective clinical trials and interventions and new therapies for rare diseases [31]. This complements research based on clinical scales, in both substantiating and graphically illustrating previous findings but also revealing patterns of behavior and experience that may be missed when using standard scales developed for other conditions. Behavior and relationships with WSS children are central in parents’ lived experiences. Finding common patterns from the parental perspective is vital both for parental understanding of the characteristics of WSS and for planning practical support and better communication with professionals in health, education and care.

## Data Availability

The data that support the findings of this study are available on request from the corresponding author. The data are not publicly available as they contain information that could compromise the privacy of research participants.

## Appendix 1

### Interview frame

Child’s age, other diagnoses, family composition.

### Section 1: Behavior and Relationships (focus of the current analysis) in each case adding: You mentioned that […] Could you go into a bit more depth /give a few examples?

1. Can you describe A’s behavior in general?
2. What is A’s behavior like at school?
3. Could you describe A’s communication style in general?
4. A’s relationships with siblings (if any) and peers Does A have any other relationships that might be important?

### Section 2: The diagnosis process

Please describe your experiences, thoughts and feelings around receiving A’s diagnosis.

How about the experiences of other members of your family around receiving that diagnosis?

Anything else you want to share in relation to the diagnosis?

Section 3: Priorities for further research, and child strengths.

What would you like to see being done in future research?

Can you describe the challenges… and then the positives about A?

## Footnotes

1 This was not a term used by parents but captures the range of characteristics across this theme.
